# Spatiotemporal evolution and driving factors of agricultural land transfer in China

**DOI:** 10.1371/journal.pone.0310532

**Published:** 2024-09-18

**Authors:** Haijiang Chen, Hong-Wai Ho, Chunli Ji, Haoqing Zheng, Songlin Zhang

**Affiliations:** 1 Business School, Shaoxing University, Shaoxing, Zhejiang, China; 2 Centre for Gaming and Tourism Studies, Macao Polytechnic University, Taipa, Macao, China; 3 School of Economics, Zhejiang Gongshang University, Zhejiang, Qiantang District, China; Sichuan Agricultural University, CHINA

## Abstract

This paper systematically analyzes the spatiotemporal evolution trends and macroeconomic driving factors of farmland transfer at the provincial level in China since 2005, aiming to offer a new perspective for understanding the dynamic mechanisms of China’s farmland transfer. Through the integrated use of kernel density estimation, the Markov model, and panel quantile regression methods, this study finds the following: (1) Farmland transfer rates across Chinese provinces show an overall upward trend, but regional differences exhibit a "U-shaped" evolution characterized by initially narrowing and then widening; (2) although provinces have relatively stable farmland transfer levels, there is potential for dynamic transitions; (3) factors such as per capita arable land, farmers’ disposable income, the social security level, the urban‒rural income gap, the urbanization rate, government intervention, and the marketization level significantly promote farmland transfer, while inclusive finance inhibits transfer, and agricultural mechanization level and population aging have heterogeneous impacts. Therefore, to achieve convergence of low farmland transfer regions to medium levels while promoting medium-level regions to higher levels, it is recommended that the government increase support for agricultural mechanization, increase farmers’ income and social security levels, and optimize marketization processes and government intervention strategies. The main contributions of this paper are (1) systematically revealing the spatiotemporal evolution patterns of China’s farmland transfer and (2) employing panel quantile regression methods to explore the heterogeneous impacts of driving factors, providing more precise and detailed empirical support for the government’s formulation of farmland transfer policies.

## 1. Introduction

China’s farmland system serves as a crucial determinant of the country’s economic and social development. The initial implementation of the household responsibility system during the early phases of China’s reform and opening-up resulted in a remarkable increase in productivity. According to Lin (1992), the rural reform measures implemented between 1978 and 1984 accounted for 48.64% of the rural economic output. Notably, the household responsibility system emerged as the primary driver, accounting for 96.40% of the overall rural reform contribution [1]. Nevertheless, China’s urbanization process and relaxed household registration system led to substantial rural-to-urban migration. This migration delayed farmland system reform and had adverse impacts on China’s economy and society.

Given the significance of farmland transfer, this paper concentrates on two primary issues: What are the current status and trends of farmland transfer across provinces in China? How can tailored strategies be developed to address the varying levels of farmland transfer in different provinces? By employing kernel density estimation, Markov models, and panel quantile regression methods, this paper reveals the spatiotemporal evolution patterns and macroeconomic driving factors of farmland transfer in China’s provinces. It offers detailed and robust answers to these two primary questions, providing solid empirical evidence that can provide government entities with a deeper understanding of the current status and anticipated future trends of farmland transfer across provinces, enabling them to devise appropriate and scientific strategies to promote transfer in each province.

The study reveals that the farmland transfer rate across China’s provinces has an overall upward trend but that significant regional disparities exist, revealing a "club convergence" phenomenon. Furthermore, key macro factors, such as per capita arable land area, the level of agricultural mechanization, and farmers’ per capita disposable income, significantly influence farmland transfer, with varying effects noted among provinces with different levels of transfer. On the basis of these findings, this paper develops a multidimensional policy recommendation framework, which includes specific development paths for provinces at various levels of farmland transfer, the creation of phased and long-term farmland transfer policies, and suggested universal policy guidelines.

The structure of the subsequent sections is as follows: Section 2 provides a literature review; Section 3 introduces the research methodology, variable selection, and data sources; Section 4 analyzes the spatiotemporal evolution characteristics of farmland transfer in China; Section 5 explores the macroeconomic driving factors of farmland transfer; and Section 6 summarizes the research conclusions and offers policy recommendations.

## 2. Literature review

The transfer of farmland management rights, referred to as farmland transfer throughout this article, is a critical component of farmland system reform in China. In recent years, rapid industrialization and urbanization have led to vigorous farmland transfer activity. Scholars have conducted extensive research on this issue, which can be categorized into three main areas: the relationship between farmland transfer and the modernization of agriculture in China, the key agents driving farmland transfer, and the factors influencing farmland transfer.

In pertinent studies regarding farmland transfer and agricultural modernization in China, Yuneng and Bo (2011) reported that the development of land transfer systems in Chinese agriculture is characterized by commercialization, centralization, and transactionalization [2]. These characteristics facilitate the redistribution of land to more efficient agricultural producers, thereby enhancing agricultural productivity through scale effects. These factors are vital for the modernization of agriculture in China (Fei et al., 2021) [3]. However, the impact of agricultural land transfer on modernization varies by region. Ren and Zhu (2023) noted that land transfer has a particularly positive effect on agricultural modernization and transformation in China’s eastern regions, whereas the impact is less pronounced in the western regions [4]. This variation is due mainly to greater industrialization and urbanization in the eastern regions, where corporate capital and agricultural technology companies are better developed. These enterprises and organizations can utilize modern technologies and management techniques to increase agricultural productivity. Additionally, government policies supporting land transfer and agricultural modernization tend to be implemented more quickly and effectively in eastern China. Second, with respect to the impact of land transfer on the ecological environment, land transfer influences the inputs of pesticides and fertilizers in agriculture. Wu et al. (2021) reported that land transfer is associated with fertilizer inputs. By consolidating land plots, land transfer can lead to more efficient and sustainable agricultural practices, thereby reducing fertilizer usage [5]. Xu et al. (2023) further found from microsurvey data of Chinese farmers that participation in arable land transfer could reduce fertilizer usage by 52.39% and pesticide inputs by 17.56% [6]. Third, land transfer affects labor migration and urbanization. The transfer of agricultural land can free up rural labor resources, encouraging rural laborers to seek better employment opportunities in cities and thus promoting labor migration (Huang et al., 2020) [7]. This migration of rural labor to urban areas reflects the ongoing increase in China’s urbanization rate (Li, 2014) [8].

Concerning the main agents influencing farmland transfer in China, scholars widely agree that the government, farmers, agricultural enterprises, and agricultural cooperatives play significant roles. To promote agricultural land transfer in China, government entities often need to reform existing land transfer systems (Fan et al., 2020) [9]. Additionally, government development strategies typically involve the integration of land transfer systems, urbanization, and the mobility of rural labor, with the government strategically guiding land mobility via an overall plan (Zheng et al., 2012) [10]. Farmers are another crucial entity because they directly participate in the decision-making process regarding the transfer of land use rights. Their willingness to transfer land directly influences the vitality of the land transfer market (Yu et al., 2020) [11]. Agricultural enterprises, which seek profits and are equipped with ample financial resources, advanced agricultural technology, and management skills, are highly productive and strongly motivated to engage in land transfer (Deininger, 2003) [12]. As China’s land transfer market has gradually matured, the proportion of agricultural enterprises participating in the land market has increased (Wu and Ma, 2022) [13]. Agricultural cooperatives—volunteer service-oriented nonprofit organizations formed by farmers—not only increase agricultural productivity and farmer incomes through collective action but also play crucial roles in the modernization of agriculture and the large-scale operation of farmland (Wen & Jiang, 2020) [14]. Cooperatives are vital in promoting agricultural land transfer by improving market access and enhancing collective bargaining power, thereby providing farmers with more stable income opportunities. This makes land transfer more attractive and beneficial to small-scale farmers (Song et al., 2014) [15]. Moreover, cooperatives can significantly reduce land transfer transaction costs by integrating the land of farmers who work away from home, facilitating large-scale transfer (Qiu et al., 2021) [16].

Promoting agricultural land transfer is crucial for sustainable agricultural development. Existing research has focused predominantly on four key areas: rural household characteristics, village conditions, the institutional environment, and other macro factors. First, since rural households are decision-making entities in agricultural land transfer, extensive research has examined the impact of their characteristics. For example, Jiang et al. (2018) investigated the influence of individual and family characteristics on agricultural land transfer [17], and Yang et al. (2021), Chen et al. (2023), and Liu et al. (2023) examined the effects of rural households’ livelihood capital, social capital, and social stratum, respectively [18–20]. Next, agricultural land transfer is characterized by economic and social attributes deeply ingrained within rural social structures. Consequently, researchers have also investigated the impact of village conditions on farmers’ agricultural land transfer behavior. Gao et al. (2022), Guo et al. (2023), Luo et al. (2019), and Hong et al. (2023) examined the effects of neighborhood dynamics, village organizational levels, village locations and topographical conditions, and kinship networks, respectively, on agricultural land transfer [21–24]. Furthermore, the institutional environment impacts transaction costs, the future expectations of the parties involved, and the cost‒benefit of agricultural land transfer. Existing research has focused primarily on four aspects: the development of the agricultural land transfer market [25], property rights characteristics [26], agricultural subsidies [27], and government intervention [28]. In addition to examining the institutional environment, researchers have explored how macrolevel factors impact agricultural land transfer, which can offer valuable insights for policy-making authorities in formulating effective measures to promote local transfer. Zhang et al. (2022) systematically investigated the impact of macrolevel factors, including population structure, economic development level, industrial structure, and fixed asset investment, on land transfer in different provinces of China [29]. Deininger et al. (2004) examined the impact of market factors and government intervention on agricultural land transfer across provinces [30]. Previous studies have also addressed the importance of other macrolevel factors, such as regional heterogeneity [31], the level of agricultural socialization services [32], digital financial inclusion [33], and economic growth and urbanization [34], to agricultural land transfer.

Existing research has thoroughly explored the socioeconomic impacts of agricultural land transfers in China, the key stakeholders involved, and both the macroeconomic and microeconomic factors influencing these transfers. These studies have provided crucial reference points for refining the research questions in this paper, designing subsequent empirical studies, and selecting variables for panel quantile regression analysis. However, areas exist in which the literature directly relevant to this paper could be improved. First, much of the current research on agricultural land transfers is based on the microperspective of individual farmers. Although some studies have examined the influence of macroeconomic factors on land transfers, these often rely on outdated data or focus solely on specific macro variables. Systematic studies investigating the impact of broader socioeconomic factors on land transfers are less common. Given the significant role that the government plays in China’s agricultural land transfers, utilizing up-to-date and comprehensive data to systematically explore the impact of macroeconomic and social variables on regional land transfers could provide essential insights for policy-making. Second, existing research methods predominantly use mean regression analysis. However, due to the substantial regional variations in land transfer practices across China, mean regression may not adequately capture the differential impacts of macroeconomic factors across various regions. This limitation can pose significant challenges for the government’s ability to implement precise, region-specific policies, potentially affecting the effectiveness of these policies. Third, although many studies have focused on exploring the causal relationships between agricultural land transfers and specific macroeconomic variables—which is valuable for understanding the mechanisms and causal links between them—the overall lack of a comprehensive understanding of the current status and future trends of land transfers limits the ability to derive valuable policy implications from the research findings. More holistic analyses that encompass both the current landscape and anticipated developments in land transfers could offer more actionable insights for policy-makers. Improving these aspects could significantly enhance the relevance and applicability of research findings in formulating targeted and effective policies for managing agricultural land transfers in China.

We focus on the government’s role as a key player in agricultural land transfer in China and explore the trends in the spatiotemporal evolution of land transfer and the macroeconomic drivers that influence such regional transfer. By clarifying these patterns, we aim to provide empirical evidence for government agencies to better understand the current status of and potential future trends in agricultural land transfer across Chinese provinces and to devise precise, scientifically grounded strategies to promote them. This paper makes the following marginal contributions compared to existing studies. (1) Data selection: This paper utilizes panel data from 30 provinces in mainland China spanning from 2005–2021. This selection provides broader coverage and more timeliness than in existing studies. Furthermore, the study period encompasses the significant policy milestone of the 2021 implementation of the "Management Measures for the Transfer of Rural Land Management Rights," facilitating a thorough examination of the evolving trends in China’s farmland transfer. (2) Research methodology: This paper employs panel quantile regression to investigate the influencing factors of farmland transfer across provinces. This approach effectively incorporates the heterogeneity of farmland transfer in different regions into the model, providing empirical support for the formulation of precise policies. (3) Research content: We organically integrate Markov models and panel quantile regression models. Doing so not only provides strategic recommendations on how provinces can promote land transfer but also uses the marginal effects of various influencing factors at different quantiles to offer strategic suggestions on how provinces with low (or medium) levels of land transfer can converge toward medium (or medium-high) levels. This approach further expands the research perspectives related to the study of agricultural land transfers. These contributions provide a richer, more detailed understanding of the dynamics at play in the land transfer process and offer valuable insights that can inform policy decisions and facilitate the targeted development of agricultural land management practices in China.

## 3. Research methods, variables and data

### 3.1 Research methods

#### 3.1.1 Kernel density estimation

Kernel density estimation (KDE) is a nonparametric estimation method that enables depicting the distribution of a random variable through a continuous density curve. By examining the distribution position, shape, spread, and polarization trend, KDE effectively reveals the evolutionary status of a random variable [24]. Considering the geographical and temporal differences in agricultural land transfer, the nonparametric nature of the KDE method allows for capturing and reflecting these variations [35]. The basic expression for KDE is as follows:

$$f\;(x)=\frac{1}{Nh}\sum_{i=1}^{N}K\;(\frac{X_{i}-x}{h})$$ (1)

In the above equation, *N* represents the sample size, *h* denotes the bandwidth, *K* represents the kernel density function, *Xi* represents independently and identically distributed observations, and *x* represents the sample mean. The kernel density function serves as a smoothing transformation function, and it is thus required to satisfy the conditions depicted in Eq (2):

$$\left\{ \begin{array}{c} {¯}_{x\to \infty }^{lim}K\;(x)=0\\ K\;(x)\geq 0\;\int_{-\infty }^{+\infty }K\;(x)=1\\ supK\;(x)<+\infty \;\int_{-\infty }^{+\infty }{K\;(x)}^{2}<+\infty \end{array}\right.$$ (2)

When the kernel density estimation (KDE) method is used, the selection of the kernel function and the bandwidth is crucial because they significantly impact the results of the KDE. This paper employs Silverman’s bandwidth selection rule, which is a commonly used method based on the dispersion of the data. This rule helps balance the bias in and variance of the estimate, making it a practical choice for achieving reliable density estimates [36]. The Gaussian kernel is chosen for the kernel function because of its proven effectiveness in practical applications, where it is smooth and closely approximates the true probability density function. This smoothness is particularly advantageous for accurately capturing the characteristics of data distributions [37]. Such precision is particularly crucial when exploring the distribution of agricultural land transfer rates. The Gaussian kernel facilitates the effective revelation of latent features and trends in the data, which is essential for understanding the dynamics and nuances of land transfer patterns. The strategic choice of Silverman’s rule and the Gaussian kernel in this study ensures that the KDE method can robustly and accurately reflect the underlying distribution of agricultural land transfer rates, providing a solid foundation for further analysis and policy formulation.

#### 3.1.2 Markov model

The Markov model is a mathematical model based on probability theory that is used to describe the process of a system transitioning from one state to another. The core characteristic of the system is "memorylessness," meaning that its future state depends on only the current state and not the path taken to reach it. This feature makes the Markov model particularly suitable for analyzing and predicting dynamic systems with time series characteristics and state transition features [38]. Agricultural land transfer is influenced not only by individual farmers’ decisions but also by macroeconomic policies, market demand, and other factors and has complex spatiotemporal evolution characteristics [39]. By defining different states of agricultural land transfer and their transition probabilities, the Markov model can be used to analyze the dynamic changes and trends in this process effectively [40]. Following the approach of Zhou et al. (2020) [41], we divide the level of agricultural land transfer in each province into four categories from low to high and then use the Markov model to explore the characteristics of positional transitions in agricultural land transfer in each province during the sample period. The specific methods are as follows.

Given a random process *{X(t)*, *t∈T}*, where the index *t* belongs to some parameter set *T* that represents different time periods and the finite states correspond to the number of states of the random variable, Eq (3) holds for all time periods *t* and all possible states *j* and *i*. Eq (3) represents the first-order Markov property, indicating that the probability of the random variable *X* being in state *j* at time period *t* depends solely on the state of *X* at time period *t-1* [42].


$$P\{X(t)=j|X(t-1)=i,\;X(t-2)=i_{t-2},\;\cdots ,\;X(0)=i_{0}\}=P\{X\;(t)=j|X\;(t-1)\}=P_{ij}$$ (3)

In Eq (3), *Pij* represents the probability of agricultural land transfer in a specific province (city) transitioning from type *i* in time period *t-1* to type *j* in time period *t*, *nij* denotes the number of occurrences where the land transfer level transitions from type *i* to type *j*, and *ni* represents the total number of occurrences of the land transfer level in type *i*. Utilizing the maximum likelihood estimation (MLE) method, we can calculate the value of *Pij* via Eq (4):

$$P_{ij}=\frac{n_{ij}}{n_{i}}$$ (4)

#### 3.1.3 Panel quantile regression model

The panel quantile regression model is a type of regression model that estimates the impact of independent variables on the dependent variable across different quantiles of the conditional distribution. Compared with traditional panel data regression models, such as fixed or random effects models, panel quantile regression provides richer and more detailed information, making it particularly suitable for scenarios in which the data distribution is uneven and contains outliers [43]. The advantages of using the panel quantile regression model in this paper include the following. First, the heterogeneous impacts of macroeconomic drivers are captured. The quantile regression method, first introduced by Koenker and Bassett (1978), has been widely used to address heterogeneity in economic data [44]. The panel quantile model extends this approach and allows researchers to explore the impact of independent variables on the dependent variable under different conditions of the distribution, thus capturing the heterogeneous effects of economic phenomena more precisely [45]. Second, the model addresses extremes and asymmetry. Data on agricultural land transfer may contain extremes or exhibit significant asymmetry, which poses a challenge for traditional panel regression models, which typically assume a uniform distribution of error terms. The panel quantile model does not require strict assumptions about the distribution of error terms, making it more robust when dealing with extreme values and asymmetric data [45]. Third, the application of the panel quantile model provides a basis for tailored policy-making, enabling research findings to provide differentiated policy recommendations [46]. In this paper, regions with lower land transfer rates may have different policy focal points than regions with higher rates. The application of the panel quantile model can provide a scientific basis for such differentiated policies. The specific model is as follows.

In the traditional quantile regression model, the *τ*th quantile function *Q*(*τ*) of the dependent variable *y* is defined as follows:

Q(τ)=inf{y:F(y)≥τ},(0<τ<1) (5)

where *F*(*y*) represents the cumulative distribution function of *y* and τ indicates the percentage of sample data below the regression line of all the sample data. In light of this, the distribution of *y* is divided into two parts on the basis of *τ*: the proportion below the quantile *Q*(*τ*) is *τ*, and the proportion above *Q*(*τ*) is (1−*τ*). Specifically, the quantile regression model for panel data can be expressed as follows:

$$Y_{it}=x_{it}^{T}\beta_{i}+{ɑ}_{i}+u_{it},(i=1,2,\ldots ,K;t=1,2,\ldots ,T)$$ (6)

where *i* represents the province and where *t* represents the year and *x*_*it*_ represents the k*×1-dimensional explanatory variables in province *i* at time *t*. *μ* is the disturbance term. *β*_*i*_ represents the k*×1-dimensional coefficients. *α*_*i*_ represents the individual fixed effects. The conditional quantile function for panel quantile regression estimation is as follows:

$$Q_{it}(\tau |x_{it},\alpha_{i})=x_{it}^{T}\beta (\tau_{q})+\alpha_{i}$$ (7)

where *τ*∈(0,1). When *τ* takes different values, solving the weighted absolute residual minimization problem can yield parameter estimates at different quantiles. The parameter *β* is obtained through the following formula:

$$\hat{\beta }={argmin}_{\alpha ,\beta }\sum_{q=1}^{Q}\sum_{t=1}^{T}\sum_{i=1}^{N}W_{k}\rho_{\tau }(y_{it}-x_{it}^{T}\beta (\tau_{q})-\alpha_{i})$$ (8)

where *ρ*_*τ*_(*u*) is the piecewise linear quantile loss function, defined as:

$$\rho_{\tau }(u)=\left\{ \begin{array}{l} u\times (\tau -1),u<0\\ u\times \tau ,\;u\geq 0 \end{array}\right.$$ (9)

In response to various limitations of panel quantile regression (Powell, 2020) [47], Koenker (2004) proposed the penalized quantile regression method with fixed effects. This method adjusts individual effects appropriately by adding a penalty term $P(\alpha )=\sum_{i=1}^{n}\left|\alpha_{i}\right|$ with a tuning parameter *λ*, effectively reducing the variance caused by *α*_*i*_ [45]. After adding the penalty term, the coefficients of the explanatory variables at quantile points can be obtained by solving the following minimization problem:

$$min\sum_{q=1}^{q}\sum_{t=1}^{T}\sum_{i=1}^{N}W_{k}\rho_{\tau k}(y_{it}-x_{it}^{T}\beta (\tau_{q})-\alpha_{i})+\sum_{i=1}^{n}\left|\alpha_{i}\right|$$ (10)

where *W*_*k*_ represents the weight coefficient. This is used to estimate the impact of quantile points *q* on the estimated coefficients.

**Fig 1:**
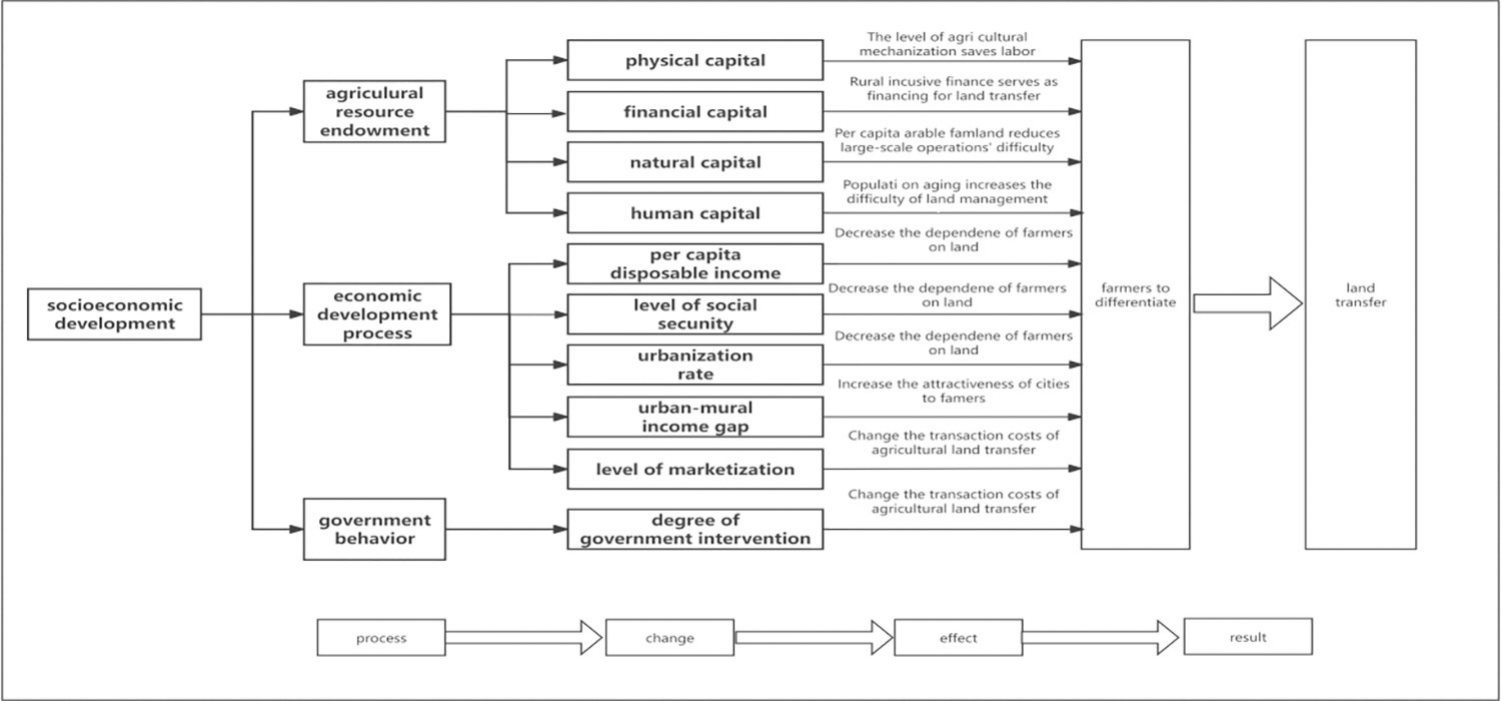
Impact mechanism of macrolevel driving factors on land transfer.

Previous research has extensively explored the macrolevel driving factors of agricultural land transfer in China, delineating three primary aspects: agricultural resource endowment [48], the economic development process [29], and government behavior (Deininger et al., 2004) [30]. Specifically, agricultural resource endowment can affect land transfer because it directly influences the difficulty of land-scale operations. Second, the economic development process can affect land transfer because it directly influences farmers’ degree of reliance on land. Finally, government behavior directly impacts the transaction costs of agricultural land transfer. Drawing on existing research and considering our research needs, this paper selects the following ten explanatory variables: agricultural mechanization, rural-inclusive finance, the per capita arable farmland, population aging, farmers’ per capita disposable income, the social security level, the urbanization rate, the urban‒rural income gap, the level of marketization, and the degree of government intervention. Fig 1 illustrates the mechanisms by which each variable affects agricultural land transfer. Variable definitions can be found in Section 3.2 Variables, and descriptive statistics for the variables are provided in Table 1 of Section 3.3 Data Sources and Statistical Description.

**Table 1:** Statistical description of variables and data sources.

Variables	Sample size	Mean	Standard deviation	Minimum	Maximum	Data source
Agricultural land transfer rate	510	0.243	0.182	0.014	0.911	*China Rural Management Statistical Annual Report*
Per capita arable farmland	510	3.886	3.712	0.507	23.578	*China Statistical Yearbook*
Rural-inclusive finance	510	1.470	0.593	0.108	7.572	*The Financial Operation Reports of Every Province in China*
Agricultural mechanization level	510	7.744	3.799	1.898	17.979	*China Statistical Yearbook*
Population aging	510	14.167	3.648	7.400	26.700	*China Statistical Yearbook*
Farmers’ per capita disposable income	510	10142.400	6243.184	1951.485	38064.23	*China Statistical Yearbook*
Social security level	510	0.132	0.077	0.015	0.309	*China Statistical Yearbook*
Urban‒rural income disparities	510	2.704	0.463	1.842	4.333	*China Statistical Yearbook*
Urbanization rate	510	0.558	0.140	0.269	0.896	*China Statistical Yearbook*
Degree of government intervention	510	0.238	0.108	0.092	0.758	*China Statistical Yearbook*
Marketization level	510	7.665	1.867	3.359	12.390	*The Report of Marketization Index in China*

### 3.2 Variables

- Dependent variable: agricultural land transfer rate. Consistent with the methodology employed by Ma et al. (2023), it is measured as the ratio of the total area of agricultural land transfer to the total area of household contracted land in China [49].
- Independent variables: In line with the research of Zhang et al. (2022) [29] and the objectives of this study, a set of 10 explanatory variables is selected.
  1. *The per capita arable farmland area*, represented as the "arable farmland area divided by the rural population" and expressed in acres per person;
  2. *Rural-inclusive finance*, represented as the "number of small rural financial institutions per rural population" [50], is measured in tens of thousands of individuals per institution. This variable is based on the research of Su et al. (2019) and takes into account the current state of rural financial development in China. Small rural financial institutions encompass rural commercial banks, rural cooperative banks, rural credit cooperatives, village banks, and rural mutual cooperatives, which cater primarily to the financial needs of rural residents;
  3. *The level of agricultural mechanization* is measured as the "total agricultural machinery power divided by arable farmland area" [30], expressed in kilowatts per hectare. This variable draws upon the methodology of Ma et al. (2023);
  4. *Population aging* is represented by the "elderly dependency ratio," in line with commonly accepted research practices;
  5. *Farmers’ per capita disposable income* is adjusted for inflation with 2005 as the base year and measured in Chinese yuan (RMB);
  6. *The level of social security* is represented as "per capita transfer income divided by per capita disposable income" among farmers. This variable is based on the studies by Behrendt and Woodall (2015) and Tirivayi et al. (2016) [51,52];
  7. *The urban‒rural income gap* is represented as "per capita disposable income among urban residents divided by per capita disposable income among rural residents," following the general academic practice;
  8. *The urbanization rate* is represented as the "urban population divided by the total population," following the general academic practices;
  9. *The degree of government intervention* is represented as "the general budgetary expenditure of local government divided by the regional gross domestic product (GDP)." This variable is based on the research by Hua et al. (2023) [53];
  10. *The level of marketization* is represented by the China Provincial Market Index compiled by Fan Gang et al., with data sourced from the China Market Index Database (https://cmi.ssap.com.cn/).

### 3.3 Data sources and statistical description

The data for this paper are sourced from multiple macroeconomic databases, including the China Rural Management Statistics Yearbook published by the Rural Cooperative Economic Guidance Department and the Policy and Reform Department of the Ministry of Agriculture and Rural Affairs, the China Statistical Yearbook released by the National Bureau of Statistics of China, the Regional Financial Operation Report issued by the provincial branches of the People’s Bank of China, and the China Provincial Marketization Index from the China Marketization Index Database. The specific data sources for each variable are detailed in Table 1.

Notably, certain variables have missing data for specific years. To impute the missing values, the study utilizes the moving average method. The data concerning agricultural land transfer are obtained from the “China Rural Management Statistical Annual Report,” with data available from 2005 onward. As a result, the study includes the years 2005–2021 for all the variables considered. The research focuses on 30 provincial-level administrative regions in mainland China, excluding Tibet. The descriptive statistics and data sources for the variables used in this study are presented in Table 1.

The statistical description in Table 1 first shows that the average rate of agricultural land transfer is 0.243, with a standard deviation of 0.182. This wide range of variation and significant standard deviation reveal the substantial heterogeneity of agricultural land transfer rates in China. Given the data characteristics, traditional parametric estimation methods might not accurately capture these distributional properties. This significantly justifies the use of Markov models and kernel density estimation, as both are nonparametric estimation methods that can more closely adhere to the actual data distribution and provide a deep understanding of the trends in China’s agricultural land transfer rates. Furthermore, the diversity in land transfer rates across Chinese provinces necessitates that policy-makers adopt differentiated strategies tailored to different levels and conditions of land transfer. The panel quantile regression model facilitates this differentiation by revealing the varying impacts of macroeconomic variables at different land transfer rates, thereby providing a basis for formulating more precise and effective policies. Second, the large standard deviations of variables such as the per capita arable land area and level of agricultural mechanization reflect significant differences in agricultural resource allocation and technological application across different regions in China. This highlights the necessity of using panel data analysis to capture the heterogeneity among regions. Third, the statistical characteristics of variables such as rural-inclusive finance, population aging, farmers’ per capita disposable income, the social security level, the urban‒rural income gap, the urbanization rate, the degree of government intervention, and the level of marketization reveal multidimensional macroeconomic factors that influence land transfer. These characteristics further validate the applicability of the panel quantile regression model in this study, enabling a detailed analysis of the impact of these factors on land transfer under varying economic conditions.

## 4. Spatiotemporal evolution of agricultural land transfer in China

### 4.1 Overview of the spatiotemporal distribution of agricultural land transfer in China

Land in China is vital for ensuring food security and serves as a form of social security for farmers to a certain extent. The Chinese government has been cautious in implementing farmland system reform measures. The reform of the farmland system is thus a prerequisite and safeguard for land transfer [54]. A thorough review of the relevant land policies reveals that there were insufficient measures in place to ensure the smooth transfer of farmland before 2008, even though farmers were gradually relying less on land due to the expansion of secondary and tertiary industries, as well as urbanization. As a result, agricultural land transfer was relatively infrequent in numerous regions before 2008. Nevertheless, transfer rates were relatively high in regions where a substantial number of laborers migrated for employment opportunities. In February 2008, the Chinese government issued the Measures for Land Registration to establish standardized procedures for land rights registration. A policy decision addressing several major issues related to rural reform and development was subsequently released by the central authorities. This decision explicitly focused on ensuring the long-term stability of land-contracting relationships. It also emphasized the need to establish management and service systems to transfer land-contracting rights and quickly create a market for such transfer. Agricultural land transfer has since been institutionalized and standardized, causing its pace to accelerate in various regions.

Table 2 provides a detailed overview of the annual farmland transfer rates and rankings for each province in China from 2005–2021, along with the overall transfer rates and rankings across all years. The data reveal that Beijing, Shanghai, Jiangsu, and Zhejiang consistently rank among the top five provinces (or municipalities) in terms of agricultural land transfer. Classified as "first-tier" regions in China, they are characterized by high urbanization rates and advanced industrial structural transformation, indicating that regional economic development is a significant macro factor influencing agricultural land transfer. In contrast, Gansu, Guizhou, Shanxi, Yunnan, and Hainan rank at the bottom in terms of agricultural land transfer rates in 2021. This could be attributed to farmers’ strong reliance on land in these five provinces. According to the relevant data from the National Bureau of Statistics in 2022, these provinces have a primary industry (agriculture) contribution exceeding 10%. Hainan ranks second, behind only Heilongjiang, with an impressive primary industry contribution of 21%. In Shanxi, the abundance of coal resources has resulted in the occupation of land by a significant number of coal mines and factories in rural areas. The high costs related to land leasing or requisition, particularly for coal mines and factories, have increased the expectations of local farmers regarding land transfer prices, thus impeding the regular development of the agricultural land transfer market in Shanxi Province. According to the analysis conducted by the Shanxi investigation team of the former Ministry of Agriculture, this factor is a key reason for the slow pace of farmland transfer in the region (http://www.moa.gov.cn/ztzl/bxwhdy/gongzdt/ 201404/t20140429_3888780.htm).

**Table 2:** Agricultural land transfer rates and rankings of provinces in China from 2005–2021.

Province	2005	Ranking	2009	Ranking	2013	Ranking	2017	Ranking	2021	Ranking	Comprehensive evaluation	Ranking
Beijing	0.119	4	0.461	2	0.484	3	0.632	2	0.642	2	0.447	2
Tianjin	0.087	6	0.132	11	0.209	16	0.485	6	0.498	7	0.277	10
Hebei	0.028	18	0.042	27	0.170	22	0.333	20	0.327	17	0.180	20
Shanxi	0.017	26	0.043	26	0.143	27	0.175	29	0.155	28	0.111	29
Inner Mongolia	0.015	29	0.106	13	0.231	14	0.372	14	0.391	15	0.223	16
Liaoning	0.020	25	0.041	28	0.160	23	0.381	12	0.325	18	0.182	19
Jilin	0.041	14	0.010	16	0.184	19	0.368	15	0.416	12	0.209	17
Heilongjiang	0.066	9	0.222	6	0.443	5	0.521	5	0.560	5	0.367	5
Shanghai	0.302	1	0.553	1	0.623	1	0.754	1	0.900	1	0.654	1
Jiangsu	0.097	5	0.294	5	0.567	2	0.615	3	0.627	3	0.431	4
Zhejiang	0.175	3	0.346	3	0.450	4	0.568	4	0.598	4	0.433	3
Anhui	0.040	15	0.114	12	0.333	7	0.455	7	0.451	11	0.286	9
Fujian	0.065	10	0.140	10	0.252	12	0.356	18	0.321	19	0.228	14
Jiangxi	0.059	11	0.104	14	0.205	18	0.359	17	0.467	9	0.233	13
Shandong	0.014	30	0.058	24	0.175	21	0.344	19	0.470	8	0.201	18
Henan	0.023	22	0.094	18	0.330	8	0.380	13	0.318	20	0.235	12
Hubei	0.025	20	0.089	19	0.265	11	0.446	8	0.373	16	0.237	11
Hunan	0.075	8	0.201	8	0.299	10	0.426	10	0.457	10	0.287	8
Guangdong	0.194	2	0.208	7	0.323	9	0.403	11	0.536	6	0.299	7
Guangxi	0.042	13	0.062	23	0.137	28	0.232	25	0.207	24	0.137	25
Hainan	0.024	21	0.023	30	0.055	30	0.103	30	0.039	30	0.049	30
Chongqing	0.055	12	0.319	4	0.383	6	0.432	9	0.413	13	0.334	6
Sichuan	0.081	7	0.151	9	0.233	13	0.367	16	0.294	21	0.227	15
Guizhou	0.034	16	0.088	20	0.210	15	0.212	26	0.167	27	0.157	24
Yunnan	0.021	24	0.065	22	0.150	26	0.208	28	0.114	29	0.114	28
Shaanxi	0.026	19	0.057	25	0.118	29	0.247	24	0.239	23	0.134	26
Gansu	0.016	27	0.029	29	0.154	25	0.259	23	0.190	26	0.131	27
Qinghai	0.015	28	0.095	17	0.178	20	0.278	21	0.253	22	0.162	22
Lingxia	0.022	23	0.072	21	0.208	17	0.277	22	0.198	25	0.158	23

Note: The data are sourced from the China Rural Management Statistical Annual Report; the shaded area represents major grain-producing provinces. The second column from the right shows the overall evaluation of farmland transfer rates over all years, and the first column from the right displays the overall ranking of farmland transfer rates for the entire period.

Notably, none of the 13 major grain-producing provinces is ranked the lowest in terms of agricultural land transfer rates. Additionally, despite its relatively high economic development level, Heilongjiang ranks fifth in the comprehensive evaluation of agricultural land transfer rates. This is due to the gradual and progressive institutional reforms in China, where the land system holds immense importance as the foundation for all other systems. Consequently, the Chinese government conducted policy pilots in major grain-producing areas before implementing reforms, granting these provinces a policy advantage in agricultural land transfer.

### 4.2 Dynamic evolution of agricultural land transfer in China

**Fig 2:**
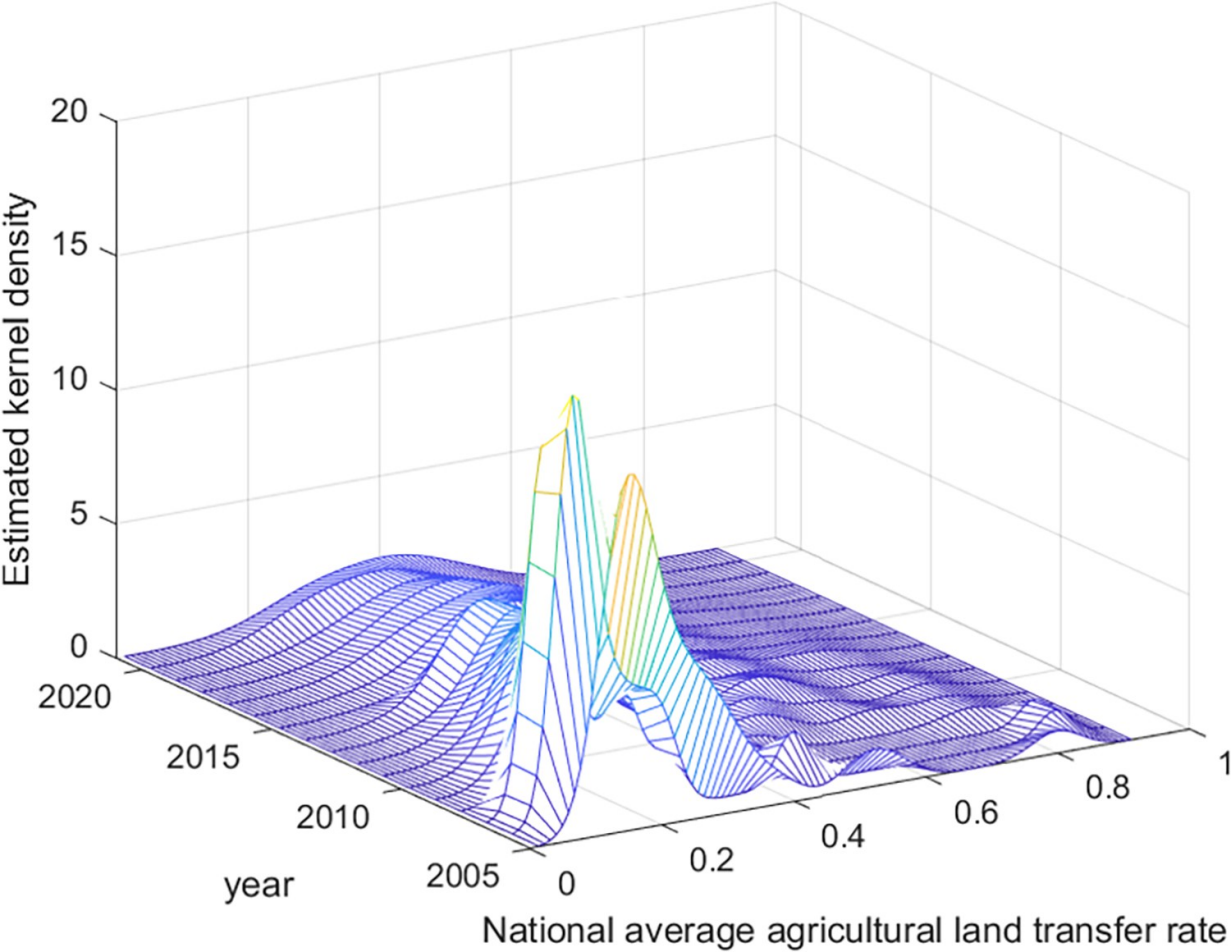
Distribution of the dynamic evolution of average agricultural land transfer rate in China from 2005 to 2021. Data source: Same as Table 2.

**Fig 3:**
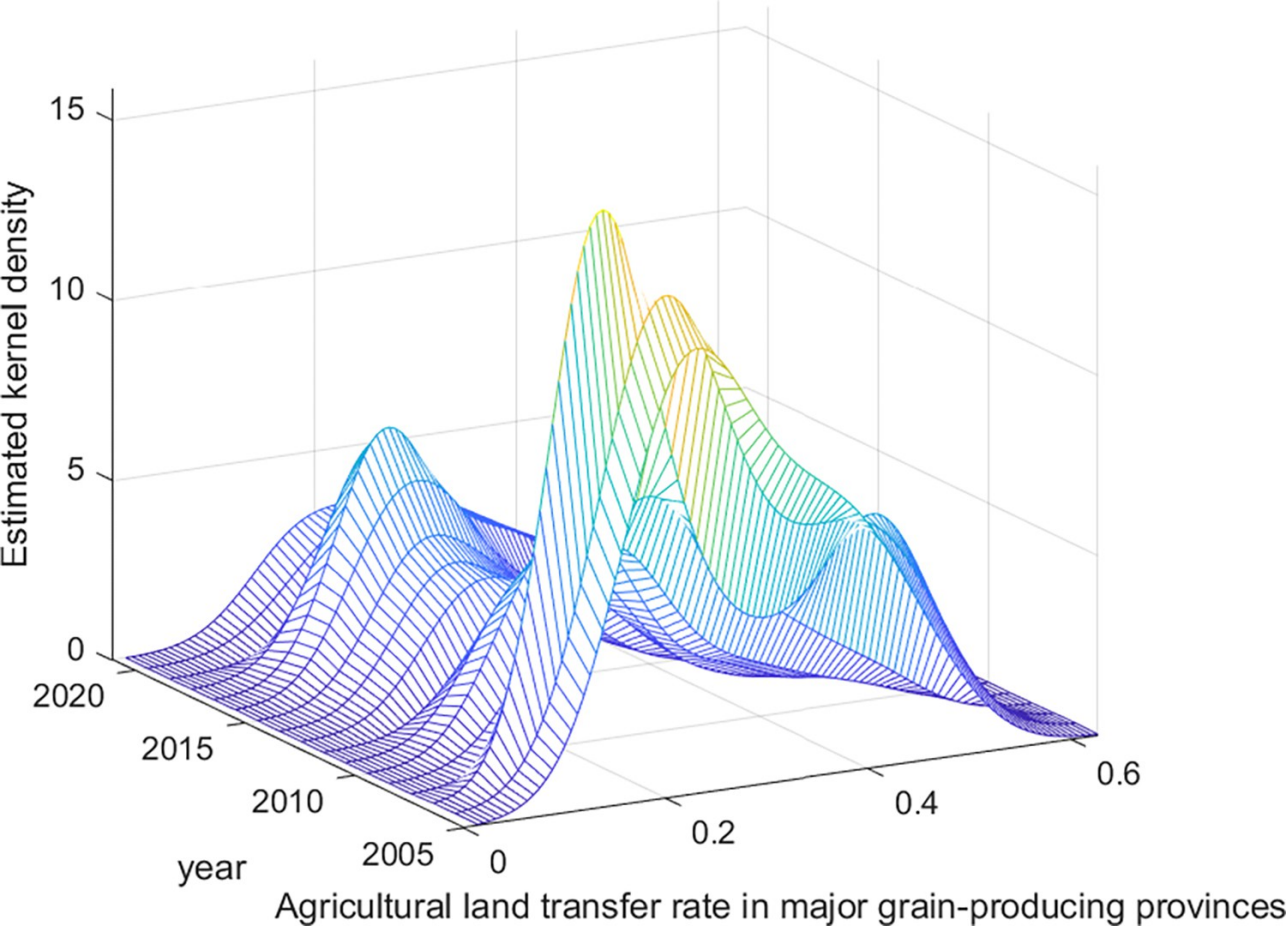
Distribution of the dynamic evolution of agricultural land transfer rates in major grain-producing provinces from 2005 to 2021. Data source: Same as Table 2.

**Fig 4:**
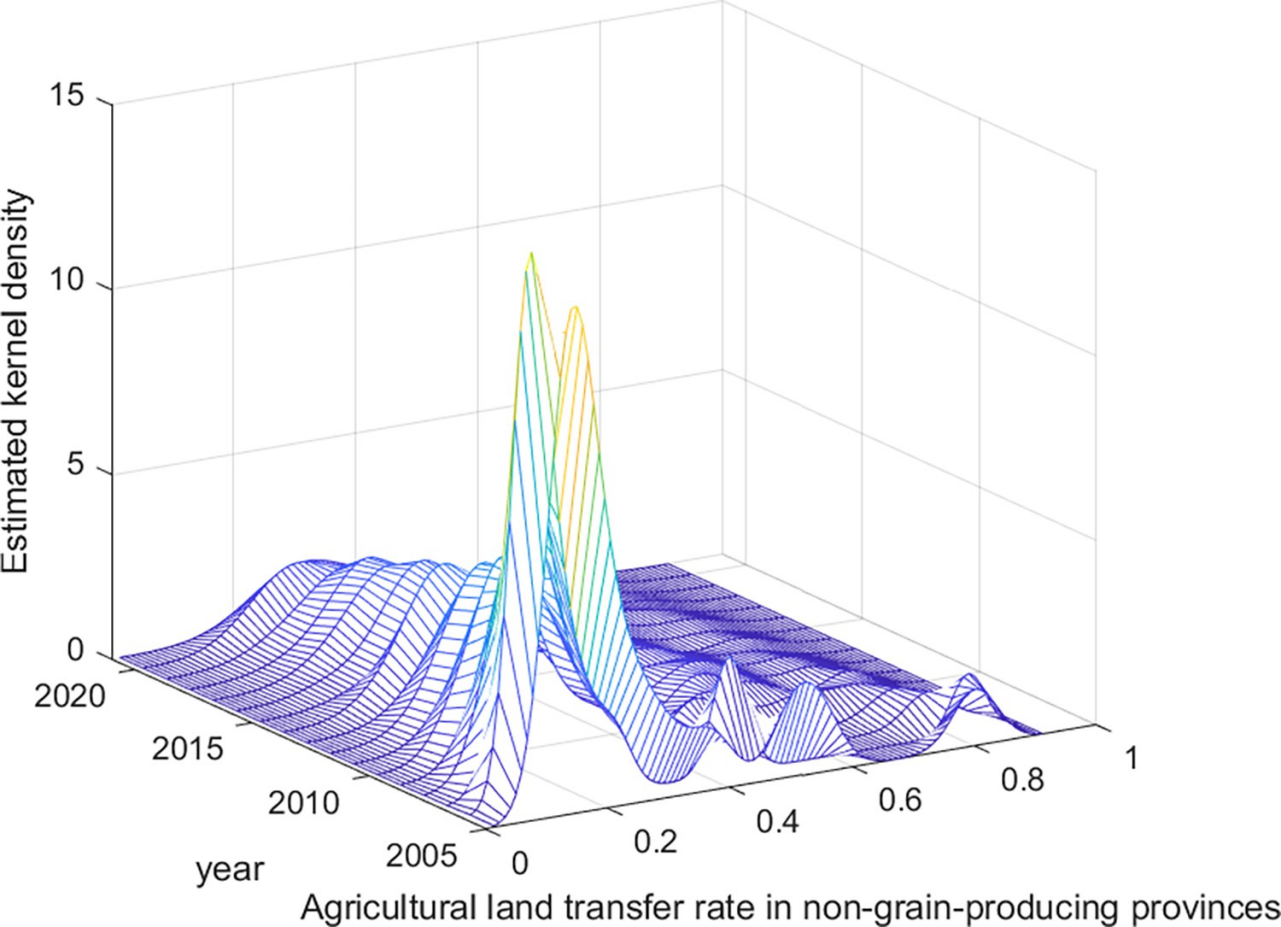
Distribution of the dynamic evolution of agricultural land transfer rates in non-major grain-producing provinces from 2005 to 2021. Data source: Same as Table 2.

The dynamic evolution of agricultural land transfer in China is further explored in this study via the kernel density estimation method. The distribution location, shape, spread, and polarization trend of agricultural land transfer at the national level, in major grain-producing provinces, and in nonmajor grain-producing provinces are examined. The findings of this analysis are presented in Figs 2–4.

First, in terms of distribution location, the average agricultural land transfer rate at the national level and in nonmajor grain-producing provinces has tended to shift toward the right, indicating overall growth in transfer rates during the observed period. Similarly, the distribution of agricultural land transfer rates in major grain-producing provinces, except for a slight leftward shift in some years, demonstrates an overall rightward movement. This pattern indicates that the average agricultural land transfer rates in major grain-producing provinces has fluctuated but shown an overall increasing trend during the observed period.

In terms of distribution shape, the distribution curves of average agricultural land transfer rates at the national level, in major grain-producing provinces, and in nonmajor grain-producing provinces consistently exhibit a transition of increasing peak height and decreasing width to decreasing peak height and increasing width. This pattern indicates that, at the national level and in major grain-producing and nonmajor grain-producing provinces, the disparities in agricultural land transfer rates initially decreased but then increased. Notably, the distribution curve of average agricultural land transfer rates in major grain-producing provinces displays a greater width than do those at the national level and in nonmajor grain-producing provinces. This suggests a greater degree of variation in average agricultural land transfer rates, specifically within major grain-producing regions.

Third, in terms of distribution spread, all three categories (nationally, in major grain-producing provinces, and in nonmajor grain-producing provinces) exhibit a distinct right-skewed tail phenomenon in the distribution curves of agricultural land transfer rates. The right-skewed tail is more pronounced in the distribution curves at the national level and in nonmajor grain-producing provinces than in major grain-producing provinces. This suggests that certain provinces in China, in both the grain-producing and nongrain-producing regions, demonstrate significantly higher agricultural land transfer rates. However, such rates at the national level and in certain provinces in nonmajor grain-producing regions are higher than those in major grain-producing regions. In particular, Shanghai, as a provincial-level municipality and a nonmajor grain-producing region, exhibited an exceptionally high agricultural land transfer rate of 90% in 2021. On the one hand, this is because Shanghai has been at the forefront of the country regarding the certification, registration, and issuance of rural land contract management rights, as well as the implementation of land management rights mortgage loans. The reform of property rights systems has provided solid institutional guarantees for farmland transfer. On the other hand, as Shanghai is a densely populated and highly developed metropolis, many of its farmers have preferred to exit agricultural production because of the abundance of nonagricultural employment opportunities and relatively low returns from farming. Consequently, they are more inclined to transfer their contracted land to large-scale farmers or new agricultural management entities, such as cooperatives [55]. In contrast, Jiangsu, the leading province in terms of the agricultural land transfer rate among major grain-producing provinces, reached a relatively high rate of 62.7% in the same year; nevertheless, this rate was significantly lower than that of Shanghai.

Finally, an analysis of the distribution curves at the national level, in major grain-producing provinces and in nonmajor grain-producing provinces reveals polarization in agricultural land transfer rates. All three categories exhibit a multimodal phenomenon, indicating the presence of multiple peaks in transfer rates. However, starting in 2007, these distribution curves gradually transition from multimodal to unimodal. This pattern indicates a weakening trend in the overall polarization of agricultural land transfer rates at the national level, in major grain-producing provinces, and in nonmajor grain-producing provinces. Table 3 summarizes the distribution dynamics and evolutionary characteristics of agricultural land transfer in China.

**Table 3:** Distribution dynamics and evolutionary characteristics of agricultural land transfer in China.

Region	Shapes of distributions	Main peak distribution shape	Distribution extensibility	Number of peaks
Nationwide	Rightward shift	Height: Rising then falling Width: Decreasing then increasing	Rightward, extended and broadened	Multimodal or unimodal
Major grain-producing provinces	Leftward shift and then rightward shift	Height: Rising then falling Width: Decreasing then increasing	Rightward, extended and broadened	Multimodal or unimodal
Nonmajor food-producing provinces	Rightward shift	Height: Rising then falling Width: Decreasing then increasing	Rightward, extended and broadened	Multimodal or unimodal

Data source: Same as Table 2.

### 4.3 Dynamic transfer paths of agricultural land transfer in China

This study employs the Markov probability transition matrix to analyze the internal flow direction and transfer locations of agricultural land transfer in China. On the basis of the magnitude of their agricultural land transfer rates, provinces across the country are categorized into four levels: I (low), II (medium–low), III (medium–high), and IV (high). The transition matrix for agricultural land transfer in each province under investigation is subsequently computed using a one-year lag as a criterion. The detailed results are presented in Table 4.

**Table 4:** Markov transition probabilities of agricultural land transfer in China from 2005–2021.

t/t+1	Ⅰ	Ⅱ	Ⅲ	Ⅳ	Observations
Ⅰ	0.7874	0.2047	0.0000	0.0079	127
Ⅱ	0.0246	0.7377	0.2377	0.0000	122
Ⅲ	0.0000	0.0583	0.7750	0.1667	120
Ⅳ	0.0000	0.0000	0.0450	0.9550	111

Data source: Same as Table 2.

Several observations can be made from Table 4. First, the average probabilities along the diagonal are greater than those off the diagonal. Among the provinces with agricultural land transfer rates classified as levels I, II, III, and IV, the probabilities of maintaining their original rankings after one year are 78.74%, 73.77%, 77.50%, and 95.50%, respectively. This suggests a relatively stable state of agricultural land transfer across different levels, indicating a club convergence phenomenon. Furthermore, the probabilities of maintaining the current status at both ends of the diagonal (78.74% and 95.50%) are greater than those in the middle of the diagonal (73.77% and 77.50%). This implies a more pronounced occurrence of the club convergence phenomenon in both low-level and high-level convergence. Second, agricultural land transfer between different levels predominantly occurs between adjacent types, with a minimal possibility of leapfrog transfer. Third, for provinces at levels I, II, and III, the probabilities of moving up one level after one year are 20.47%, 23.77%, and 16.67%, respectively. This highlights the varying challenges faced by provinces at different levels in promoting agricultural land transfer. Consequently, future policies aimed at facilitating agricultural land transfer should be specifically tailored to these different levels. In addition, provinces classified as levels II, III, and IV are at risk of downward mobility. The probabilities of moving down one level after a year are 2.46%, 5.85%, and 4.50%, respectively. Despite the relatively low probabilities, those provinces should exercise caution regarding the potential for downward mobility, as the advancement of agricultural land transfer plays a crucial role in efficiently allocating labor and land resources in the context of industrialization and urbanization.

## 5. Analysis of macrolevel driving factors of agricultural land transfer in China

From the analysis of the Markov probability transition matrix, it is evident that the level of farmland transfer affects the subsequent difficulty of further promoting farmland transfer in a region. Decision-making government entities need to consider "policy implementation based on levels." To delve deeper into this heterogeneity, this paper employs panel quantile regression to investigate the macroeconomic driving factors of farmland transfer in China. Panel quantile regression can capture heterogeneous effects under different quantile conditions, which is crucial for formulating targeted policies. To maintain consistency with the Markov probability transition matrix analysis mentioned earlier, this section utilizes the panel quantile model to explore the driving factors of farmland transfer across provinces, selecting the Q25, Q50, and Q75 quantiles for the regression results. Analyzing different quantiles reveals the heterogeneous characteristics of farmland transfer in low-, medium-, and high-level regions, enabling us to provide more targeted policy recommendations for each region. Table 5 presents the specific regression results.

**Table 5:** Quantile regression results for the driving factors of agricultural land transfer in China.

Explained variable: Agricultural land transfer rate						
Explanatory variables	Model 1 (Q25)		Model 2 (Q50)		Model 3 (Q75)	
	Coefficient	Standard error	Coefficient	Standard error	Coefficient	Standard error
Per capita arable farmland	0.008***	0.001	0.006***	0.001	0.008***	4.38e-04
Rural-inclusive finance	-0.035***	0.003	-0.032**	0.016	-0.051***	0.001
Agricultural mechanization level	0.001***	3.30e-04	-0.003***	4.24e-04	0.002***	0.001
Population aging	0.002***	1.94e-04	9.43e-05	4.49e-04	0.006***	2.35e-04
Farmers’ per capita disposable income	2.03e-05***	2.48e-07	1.92e-05***	1.91e-06	1.73e-05***	2.33e-07
Social security level	0.083***	0.022	0.247**	0.113	0.175***	0.005
Urban‒rural income disparities	0.029***	0.003	0.021***	0.002	0.037***	0.003
Urbanization rate	0.052***	0.017	0.106**	0.042	0.424***	0.008
Degree of government intervention	0.138***	0.015	0.096***	0.012	0.122***	0.006
Marketization level	0.018***	8.90e-04	0.027***	0.004	0.022***	0.001
Sample size	510		510		510	

Note

** denotes significance at the 5% level, and

*** denotes significance at the 1% level.

In the regression results presented in Table 5, the impacts of various factors clearly exhibit significant heterogeneity across provinces with different levels of farmland transfer. At the Q25, Q50, and Q75 quantiles, the directions of the effects of most factors are generally consistent; however, their significance and magnitude vary significantly. This fully reflects the complexity of the mechanisms influencing farmland transfer.

First, the per capita arable farmland, urban‒rural income disparities, and degree of government intervention positively influence farmland transfer in various provinces, but their marginal effects exhibit a U-shaped trend across different quantiles. These three factors promote farmland transfer in provinces for the following reasons: Per capita arable farmland is closely related to regional land abundance. According to Cheng et al. (2022) [56], areas with greater abundance can achieve plot consolidation through farmland transfer, reaching the threshold of "economies of scale" more easily, thus yielding greater benefits from farmland transfer. The urban‒rural income gap leads farmers to seek work in cities, creating demand for land reallocation, which is the most significant practical condition for large-scale farmland transfer in China [57]. Reasonable government intervention can guide orderly farmland transfer and optimize allocation [58]. Furthermore, the U-shaped trend of these three factors at different quantiles may be due to the following: Regarding the per capita arable farmland area, provinces with low levels of farmland transfer have a greater proportion of agriculture and lower modernization levels. Increasing the per capita arable farmland is conducive to scale management and transfer. Provinces with medium-to-high levels of transfer have mature markets and low costs; as a result, increasing the per capita arable land in those provinces can leverage market advantages for scale allocation. Provinces with intermediate levels of transfer are somewhere in the middle; their modernization is relatively high, and while there is strong supply and demand, market development is insufficient, weakening the marginal promotion effect of increasing the per capita arable farmland. The U-shaped trend in the urban‒rural income disparities may be related to the degree of part-time farming. In provinces with low farmland transfer levels, local nonagricultural employment opportunities are scarce due to lower economic development. Therefore, nonagricultural employment often requires long-distance migration, leading to higher opportunity costs for part-time farming. In provinces with medium-to-high levels of farmland transfer, which are typically located in economically developed regions of China (where the correlation coefficient between per capita GDP and the farmland transfer rate is as high as 0.832), as nonagricultural employment opportunities are abundant and stable, part-time farming is not necessary as a risk diversification strategy. Under significant urban‒rural income disparities, these regions tend to transfer land. Conversely, in provinces with intermediate transfer levels, there are more local nonagricultural opportunities than in low-transfer regions, but nonagricultural employment is not as stable as that in medium- to high-transfer regions. Hence, farmers in these areas might choose part-time farming as a risk diversification strategy, making urban‒rural income disparities less effective in promoting farmland transfer. A possible reason for the U-shaped trend in government intervention is that provinces with low levels of farmland transfer have low starting points and more room for improvement. Government intervention can quickly establish infrastructure and institutional frameworks, effectively reducing the transaction costs associated with information asymmetry and increasing farmers’ confidence in land transfer through policy guarantees. In provinces with medium-to-high levels of transfer, mature market mechanisms provide a solid foundation for government intervention. There, the government can further promote transfer by improving institutions and providing advanced services, and these regions’ demonstration effects also encourage increased government support. In contrast, provinces with intermediate levels of farmland transfer may face diminishing marginal returns and structural barriers, and they are in a transitional phase from government-led to market-led transfer. This makes the effect of government intervention there less pronounced than at both ends of the spectrum.

In addition, rural-inclusive finance has inhibited land transfer in various provinces. Schultz argues in "Transforming Traditional Agriculture" that the agriculture industry inherently possesses the capacity for self-sufficiency, and the widespread poverty among farmers in developing countries is due primarily to a lack of investment [59]. Rural-inclusive finance helps rural households provide financial services for their agricultural production, easing their financial constraints. This indirectly affects farmers who intend to transfer their land due to financial limitations, reducing the potential for achieving economies of scale in land investments. As a result, this diminishes the motivation of contracted households to transfer their land and thus hinders land transfer across different provinces due to the influence of rural-inclusive finance.

Third, agricultural mechanization and population aging are observed to positively contribute to land transfer at the 25th and 75th percentiles. However, at the 50th percentile, agricultural mechanization has a restraining effect, whereas the impact of population aging is not significant. One plausible explanation for this finding is that agricultural mechanization serves as a labor-saving technology. Its advancement positively affects labor savings for farming households and facilitates the expansion of large-scale land operations. On the other hand, population aging has noticeable adverse effects on agricultural production. When the data from Tables 2 and 5 are cross-referenced, it becomes apparent that provinces with lower land transfer rates exhibit lower economic development and limited nonagricultural job opportunities. In fact, the development of agricultural mechanization expedites the differentiation of local farmers, with some seeking off-farm employment and others expanding their operations through land transfer [60]. As young and middle-aged laborers migrate for work, elderly individuals are unable to handle intensive agricultural tasks alone. Consequently, agricultural mechanization and population aging contribute to the promotion of land transfer in these provinces. For provinces with moderate land transfer rates, the availability of nonagricultural job opportunities allows farmers to balance farming and off-farm employment. The development of agricultural mechanization enables them to maximize income through diversified operations. The partial involvement of young and middle-aged laborers in agricultural production also allows elderly individuals to provide supplementary support in crop maintenance. Therefore, agricultural mechanization discourages land transfer in these provinces, and population aging has a minimal effect on land transfer. For provinces with higher land transfer rates, it is evident from Table 2 that they tend to be economically developed, with farmers exhibiting reduced reliance on land. This allows elderly individuals to sustain themselves without relying on land for their livelihood in retirement. Moreover, progress in agricultural mechanization empowers farms to undertake larger-scale operations. As a result, both agricultural mechanization and population aging contribute to the promotion of land transfer in these provinces.

Fourth, the per capita disposable income, urbanization rate, and marketization level positively influence the farmland transfer rate across provinces. The per capita disposable income and urbanization rate reduce farmers’ dependence on land, thereby promoting farmland transfer [61]. The marketization level effectively reduces the institutional costs of reaching farmland transfer agreements, thus facilitating farmland transfer across provinces [62]. Furthermore, considering the marginal effects of these influencing factors on provinces with different levels of farmland transfer, the per capita disposable income clearly converges across provinces with different farmland transfer levels. In contrast, the urbanization rate and marketization level exacerbate the disparities between provinces with different levels of farmland transfer. The convergence effect of farmers’ per capita disposable income on provinces with different levels of farmland transfer may be due to the high correlation between farmland transfer rates and economic development levels. Provinces with low transfer levels often face higher migration costs for nonagricultural employment. Increasing farmers’ per capita disposable income lowers the migration cost barrier for farmers in these provinces, increasing their ability to find nonagricultural jobs [63]. This accelerates the farmland transfer process in these regions, narrowing the gap with provinces that have higher farmland transfer levels. The reason that the urbanization rate and marketization level exacerbate the disparities between provinces with different levels of farmland transfer may be that provinces with higher transfer levels have already established relatively mature land transfer mechanisms. Consequently, further development in urbanization and marketization can more effectively activate land resources, producing a hierarchical effect on farmland transfer [64].

Fifth, the level of social security positively promotes farmland transfer across all provinces, but its marginal effects exhibit an inverse U-shaped trend at the Q25, Q50, and Q75 quantiles. In China, land possesses a certain degree of social security function [65], and increasing social security levels weakens this function of land, thereby reducing farmers’ dependence on it and promoting farmland transfer. The inverse U-shaped trend of social security levels at different quantiles may result from the fact farmers still rely heavily on land when the social security level is low. While improving social security can reduce this dependence, the marginal effect on promoting farmland transfer is limited due to the immaturity of other conditions for transfer [65]. However, when the social security level reaches a certain point, farmers’ dependence on land has already significantly decreased, and the main barriers to transfer are no longer related to social security. At this stage, further improving social security will gradually have a diminishing marginal effect on promoting farmland transfer [66].

In addition, the previous discussions on land transfer in China, which employed kernel density estimation and Markov transition probability analysis, highlighted the rising disparities in land transfer levels among regions in China. Provinces at different levels of land transfer face the possibility of upward transitions but also the risk of downward transitions. Considering the urgent need for responsive policies and practical feasibility (the probabilities of upward and downward transitions), this study examined how provinces could converge from low to moderate levels of land transfer, as well as how moderate levels of land transfer could further advance toward high levels. Combining the marginal effects of various influencing factors from Table 5 with the potential for policy intervention, the convergence of land transfer levels from low to moderate among the relevant Chinese provinces is facilitated by increasing the per capita arable farmland area, agricultural mechanization level, per capita disposable income of farmers, and degree of government intervention (indirect intervention). In particular, the per capita arable farmland area, which is closely linked to regional arable farmland resources, can also be increased through further migration of rural populations. Additionally, the increase in farmers’ per capita disposable income, improvements in social security, and the promotion of marketization contribute to the convergence of relevant provinces from moderate to higher levels of land transfer.

## 6. Conclusions and discussion

### 6.1 Research conclusions

This study comprehensively utilizes kernel density estimation, Markov models, and panel quantile regression methods to explore the spatiotemporal evolution and macroeconomic driving factors of farmland transfer across 30 provinces in China from 2005–2021 and construct a multidimensional policy recommendation framework. The main conclusions are as follows: (1) Spatiotemporal evolution characteristics: Between 2005 and 2021, the overall farmland transfer rate across provinces in China showed an increasing trend, indicating increasing transfer activity. However, significant regional differences exist, exhibiting a club convergence phenomenon, which reflects the layered stability of farmland transfer development. This finding provides a new perspective for understanding the complexity and diversity of farmland transfer, highlighting the necessity of formulating tailored policies according to local conditions. (2) Macroeconomic driving factors: The results of the panel quantile regression indicate that key macroeconomic factors, such as the per capita arable land area, the level of agricultural mechanization, and farmers’ per capita disposable income, have significant impacts on farmland transfer. Importantly, these factors exhibit differentiated effects across provinces with varying levels of transfer. For example, the level of agricultural mechanization has a more pronounced effect on regions with low transfer rates, whereas the degree of marketization has a greater influence on regions with high transfer rates. This finding provides empirical support for the precise formulation of policies to promote farmland transfer.

On the basis of the above research conclusions, this paper constructs a multidimensional policy recommendation framework: (1) Providing specific paths for transition involves helping low-transfer provinces transition to medium levels and medium-transfer provinces progress to high levels. For low-transfer provinces (e.g., Hainan Province and Yunnan Province), the primary focus should be on improving agricultural infrastructure. For medium-transfer provinces (e.g., Fujian Province and Sichuan Province), the emphasis should be on enhancing marketization and optimizing government guidance. (2) Formulating short-term and long-term farmland transfer policies: On the basis of the predictions from the Markov model, this paper suggests focusing on supporting low-transfer regions in the short term by improving infrastructure and increasing mechanization to increase the transfer potential. In the medium term, attention should be given to transforming medium-transfer regions, with a focus on optimizing the market environment and government policies. In the long term, efforts should be made to perfect the farmland transfer market mechanisms and relevant legal systems and cultivate new types of agricultural management entities to promote overall farmland transfer. (3) Universal policy orientation: Important measures that should be applied across all provinces and stages include continuously increasing farmers’ incomes, improving social security, narrowing the urban‒rural income gap, and enhancing marketization, all of which create a favorable macro environment for farmland transfer.

### 6.2 Discussion

We explore the evolutionary trends and macroeconomic drivers of agricultural land transfer in China from both spatial and temporal perspectives. However, we improve on existing studies in terms of data timeliness and research methods and extend beyond merely examining the macroeconomic factors promoting land transfer in various provinces to further study how provinces at different levels of land transfer might converge. However, we are constrained by data availability. This research is focused primarily on the provincial level, which, while providing macrolevel insights, may obscure differences in land transfers at the municipal, county, or even village level. Future research could aim to analyze these processes at more granular levels of administrative divisions to offer more refined and practical recommendations. Moreover, although we analyze and discuss the mechanisms influencing land transfer across different regions, we do not conduct specific mechanism tests in the empirical section due to data limitations. Therefore, as more comprehensive data become available in the future, the deeper underlying mechanisms of agricultural land transfer at the macro level in China will require further exploration and research.

## Supporting information

S1 Data (XLSX)
